# Combating Antimicrobial Resistance: Innovations and Strategies

**DOI:** 10.3390/microorganisms13081869

**Published:** 2025-08-11

**Authors:** Vasiliki Koumaki

**Affiliations:** Department of Microbiology, Medical School, University of Athens, 11527 Athens, Greece; vkoumaki@med.uoa.gr

Antimicrobial resistance (AMR) is an increasingly prevalent global health problem that undermines the efficacy of critical antimicrobial agents, including antibiotics, antivirals, antifungals, and antiprotozoals. The excessive and inappropriate use of these medicines in human healthcare, agriculture, and animal husbandry has significantly hastened the emergence and spread of resistant pathogens [1]. A global response is needed to combat AMR, which includes developing new antimicrobial agents, enhancing antimicrobial stewardship, and understanding resistance mechanisms. This Special Issue features research and review articles that contribute to our knowledge of AMR in clinically important bacterial pathogens using a One Health perspective.

The vulnerability of the elderly population to resistant infections is critically examined in the study by Theodorakis et al., who highlight how biological and clinical factors, such as multimorbidity, immunosenescence, and frequent healthcare exposure increase susceptibility to multidrug-resistant pathogens, including MRSA, VRE, and CRE [2]. As traditional antibiotics become less effective, new therapeutic approaches, such as next-generation antibiotics and innovative therapies like bacteriophage therapy, are being investigated. Molecular diagnostics, such as those that improve the speed and precision of pathogen detection, enable targeted treatment. Preventive strategies focus on developing vaccines utilizing multi-epitope and nanoparticle technologies. Antimicrobial stewardship programs are important in optimizing antibiotic use and preventing resistance. In addition, artificial intelligence techniques, including machine learning, support diagnostics, resistance prediction, and the development of new therapies and vaccines are also crucial. An integrated multidisciplinary strategy combining these innovations is essential to combat antibiotic resistance in the elderly population.

AMR’s ecological profile is described by Banerji et al., who offer a perspective on how resistance traits are entangled with harmful algal blooms (HABs) [3]. AMR is a public health problem and also modifies microbial interactions and ecosystem functions. HAB cyanobacteria often possess AMR traits that may influence bloom timing, persistence, and toxicity. These traits can be spread through genetic exchange, biofilm formation, and environmental stressors. AMR can also affect food webs by affecting microbial traits that mediate interactions with other organisms. This review warns of carrying out integrated environmental management approaches among AMR and HABs. Potential solutions involve advanced genetic and ecological tools, including phages and microbial community manipulation. The need for interdisciplinary research to understand the ecological consequences of AMR is very important.

The food production chain is another clinical front in the battle against AMR, as underscored by the study by Tanzin et al. In their paper, the authors discussed the prevalence of resistance of *E. coli* in broiler meat from markets in Chattogram, Bangladesh [4]. A total of 570 samples were tested, from which half were positive for *E. coli* and three-quarters were resistant to colistin, a last-resort antibiotic. Antimicrobial susceptibility tests demonstrated a high resistance to sulphamethoxazole–trimethoprim, tetracycline, and ampicillin. Most isolates were multidrug-resistant, and some of them were resistant to all tested antibiotics. The study also identified the mcr-1 gene, responsible for colistin resistance, in all phenotypically colistin-resistant isolates. This resistance likely stems from the overuse of antibiotics in farming, which poses serious public health risks due to potential transmission through the food chain. These findings demonstrate the urgent need for antimicrobial usage and improved hygiene practices to limit resistant bacteria.

The search for effective alternatives to traditional antibiotics is at the heart of the review by Ma et al., who discuss the global crisis of AMR caused by the overuse and misuse of antibiotics [5]. Despite increased awareness, current AMR strategies are still not enough. The urgent need for novel antimicrobial strategies is emphasized in this article. It also provides an analysis of CRISPR/Cas-based systems but also presents emerging tactics such as antimicrobial polysaccharides, compounds that interfere with bacterial energy production, nano-based antimicrobial agents, and ribosomal protein antimicrobials. The authors refer to theoretical advancements and methods, exploring the potential for more biomedical applications and their role in future treatment strategies.

Region-specific surveillance and epidemiological mapping are equally essential, as shown in the study by Nirca et al. [6], who present a molecular epidemiology study of carbapenem-resistant Gram-negative bacteria in Moldova. Over 11 months, isolates were collected and analyzed for phenotypic and genotypic resistance, as well as phylogenetic relationships. The study identified several phylogenetic clusters of carbapenem-resistant *Klebsiella pneumoniae*, *Acinetobacter baumannii*, and *Pseudomonas aeruginosa* isolates, as well as some less common species and sequence types. A phylogenetic relationship to characterize isolates from the neighboring country of Ukraine was confirmed. Identified carbapenemase genes included blaKPC-23, blaOXA-72, and blaGES-11 in *A. baumannii*, blaKPC-3, blaNDM-1, and blaOXA-48 in *K. pneumoniae*, as well as blaVIM-2 in *Pseudomonas aeruginosa*. Several isolates with very high genomic resemblance further support the hypothesis of regional transmission events driven by several evolutionary successful clonal lineages. The findings underscore the importance of continued surveillance and regional collaboration to monitor and combat the spread of carbapenem-resistant pathogens.

The clinical relevance of resistant infections in the pediatric population was examined by Giormezis et al. [7], who investigated *Staphylococcus aureus* isolates responsible for skin and tissue infections in children and adolescents within the Western part of Greece. This research focused on biofilm formation, antimicrobial resistance, bacteriophage K activity, and genetic factors of resistance, involving mainly adhesins and toxins. The study highlights the importance of understanding the clinical relevance of these infections and the necessity to recognize the genetic factors contributing to virulence and resistance in *Staphylococcus aureus* strains in the young population. The elevated bacteriophage K susceptibility demonstrated is interesting in terms of exploring other therapeutic strategies against antibiotic-resistant *S. aureus*.

A retrospective, single-center study by Bagnaso et al., taking place over 17 years in Italy, investigated the etiology and antibiotic resistance of bacterial strains isolated from urinary tract infections (UTIs) in infants under six months of age [8]. An important finding was the increased prevalence of antibiotic resistance among Gram-negative bacteria, particularly to oral antibiotics like amoxicillin–clavulanate and ciprofloxacin. The study emphasized that both the prevalence of microorganisms and antibiotic susceptibility demonstrate variations, which require long-term studies to describe and analyze these patterns in young infants with UTIs.

In conclusion, AMR remains one of the most serious global health problems. The studies presented in this Special Issue show the impact of AMR, from its clinical burden in different populations to ecological effects on natural systems and food production. Solutions, like advanced molecular diagnostics, the development of next-generation antibiotics, and innovative vaccines, must be urgently provided. Epidemiological studies showed the importance of surveillance, particularly in regions with a high prevalence of carbapenem-resistant and multidrug-resistant bacterial infections. The environmental dimension of AMR leads to the need to integrate ecological perspectives and public health strategies. We need to collaborate and share knowledge not only to preserve the effectiveness of current treatments but also to ensure the prevention of infectious disease dissemination for future generations.
